# Host Defense and the Airway Epithelium: Frontline Responses That Protect against Bacterial Invasion and Pneumonia

**DOI:** 10.4061/2011/249802

**Published:** 2011-09-22

**Authors:** Nicholas A. Eisele, Deborah M. Anderson

**Affiliations:** ^1^Department of Veterinary Pathobiology, University of Missouri, Columbia, MO 65211, USA; ^2^Department of Molecular Microbiology and Immunology, University of Missouri, Columbia, MO 65211, USA; ^3^The Laboratory for Infectious Disease Research, University of Missouri, Columbia, MO 65211, USA

## Abstract

Airway epithelial cells are the first line of defense against invading microbes, and they protect themselves through the production of carbohydrate and protein matrices concentrated with antimicrobial products. In addition, they act as sentinels, expressing pattern recognition receptors that become activated upon sensing bacterial products and stimulate downstream recruitment and activation of immune cells which clear invading microbes. Bacterial pathogens that successfully colonize the lungs must resist these mechanisms or inhibit their production, penetrate the epithelial barrier, and be prepared to resist a barrage of inflammation. Despite the enormous task at hand, relatively few virulence factors coordinate the battle with the epithelium while simultaneously providing resistance to inflammatory cells and causing injury to the lung. Here we review mechanisms whereby airway epithelial cells recognize pathogens and activate a program of antibacterial pathways to prevent colonization of the lung, along with a few examples of how bacteria disrupt these responses to cause pneumonia.

## 1. Introduction

 Host defense in the mammalian lung relies heavily on innate immune mechanisms that prevent invasion of pathogens. The airway epithelium is the front line defender of the lung which signals recruitment and activation of effector cells to kill invading pathogens and provides a physical barrier loaded with antibacterial compounds. Bacteria that successfully penetrate the epithelium must have the capability to evade these mechanisms, which typically means they avoid recognition and killing by both the effector cells of the innate immune system and the antimicrobial mechanisms in the epithelium. Pneumonia is a consequence of lung colonization, pathogen-induced injury to the epithelium, sustained activation of inflammation, and overactivation of tissue repair mechanisms. Furthermore, vascular leakage and edema are caused by these host responses, allowing the pathogen to gain access to the blood, where it may spread systemically and cause sepsis. Bronchopneumonia is characterized by focal areas of congestion of the parenchyma by bacteria, inflammatory cells, and fibrin while lobar pneumonia is defined by a single area of congestion that takes up a larger portion of a lung lobe. Interstitial pneumonia involves congestion in the surrounding vasculature and is typically the result of overactive recruitment of inflammatory cells. In this paper, we will discuss how airway epithelial cells orchestrate innate immune responses in the lungs in order to limit invasion of bacterial pathogens, mediate tissue repair, and prevent pneumonia. 

The airway epithelium can be subdivided into bronchial and alveolar epithelial cells, which are polarized cells that share function in providing both a physical barrier and antimicrobial activity. The bronchial epithelial and goblet cells line the large airways, and these cells regulate ion exchange, mucin production, inflammation, and repair responses [1]. These cells form a physical barrier, connected by tight junctions, adherens junctions, and desmosomes that are relatively impermeable [2]. Similarly, the alveolar epithelium, composed of two distinct cell types, produces antibacterial compounds such as surfactant, initiates and terminates inflammation, and regulates gas exchange to provide oxygen to the body. Resident alveolar macrophages and occasionally dendritic cells are also found in the alveolar epithelium and are key mediators of innate and adaptive immunity. Type I alveolar epithelial cells function primarily in facilitating gas exchange, but they also comprise a large portion of the impermeable barrier and can sense and respond to microbial products. Type II alveolar epithelial cells, also called type II pneumocytes, function as defenders of the airway through secreting antimicrobial products, sensing pathogenic invasion, and producing cytokines and chemokines that both activate and deactivate inflammation. In addition, type II cells can differentiate into type I cells and secrete repair enzymes upon damage to the epithelium. 

Mucins are continuously secreted by intraepithelial goblet cells and are composed of large glycoproteins that cross-link to form a structural barrier [1]. This property causes small and large particles, such as proteins and whole cells, to become trapped. Mucus contains a variety of antimicrobial compounds including IgA, collectins, and defensins which are regulated by the transcription factors NF-*κ*B and Sp-1 [2]. Microbes and other particulate material are pumped outward through the action of the mucociliated bronchial epithelium, a process that requires calcium transport, and serves as an environment for the activity of antimicrobial compounds [3]. Activation of NF-*κ*B following signaling from the sentinel toll-like receptors (TLRs) induces epithelial cells to increase production of these compounds as well as proinflammatory cytokines which in turn also induce increased production of mucin. Together, these processes result in concentrated antimicrobial products that are broadly effective against invading microbes.

Type II pneumocytes secrete surfactants on the apical side of the cell which are fatty acids that elicit similar functions as mucins. Four surfactant-associated proteins, SpA-D, are also produced by type II cells that function to agglutinate microbes to facilitate their clearance. Surfactant protein B has antimicrobial activity against bacteria by enhancing the phagocytic function of alveolar macrophages [4]. A number of antimicrobial products including *β*-defensins, lipocalin, and nitric oxide, as well as complement protein C3 and interferon are secreted into the surfactant layer (Table 1). Lipocalin chelates Fe^+3^, limiting the access of bacteria to essential iron thereby stunting microbial growth [5]. *β*-defensins and nitric oxide provide direct killing of bacteria. *β*-defensins are small cationic antimicrobial peptides (CAMPs) with bactericidal activity and are attracted to the negative charge of the bacterial membrane. To maintain adequate concentrations of these peptides in the surfactant layer they are continuously secreted [6]. Type II epithelial cells also secrete repair enzymes, such as fibrinogen, on the basolateral face [7]. They quickly respond to changes in osmotic stress thereby able to sense nanomolar concentrations of bacterial pore-forming toxins. This allows cells to activate an inflammatory response long before the toxin is lethal to the cell [8].

Resident alveolar macrophages are phagocytic cells with distinct properties and initiate recruitment of inflammatory cells, such as neutrophils, as well as present antigen to cells of the adaptive immune system [9]. Uptake of pathogens by alveolar macrophages can be stimulated through antibody or complement opsonization, both of which are produced by airway epithelial cells and are also present in the blood. Recognition of intracellular or extracellular bacteria by pattern recognition receptors (PRRs) expressed by alveolar epithelial cells and macrophages stimulates an increase in the production of antimicrobial compounds, complement, cytokines, and chemokines which recruit effector cells such as neutrophils, monocytes, and dendritic cells, as well as T and B cells (Table 1) [10]. Inflammatory monocytes and neutrophils, however, must not reside in the lung long term because they produce and secrete cytotoxic molecules that will injure the delicate type I cells. Instead, polarized secretion of cytokines and chemokines by type I and II cells promotes recruitment, adherence, and transepithelial migration of inflammatory cells to fight infection, which is subsequently downregulated to limit their damage to the epithelium [11]. 

Bacterial pathogens penetrate the epithelium either through its disruption or by directly invading the airway epithelial cells. Disruption can be achieved by the induction of apoptosis or the use of bacterial exotoxins that directly lyse cells [12]. The use of toxins to promote penetration of epithelial barriers triggers injury responses that activate inflammation independent of pathogen recognition. In many cases, pathogens delay or prevent repair of the epithelium and these injury responses exacerbate lung congestion and accelerate disease [13]. In addition, some opportunistic pathogens derive help in crossing the epithelium through coinfection with viruses that impair mucociliary function, thereby allowing disruption of the epithelial barrier. 

Many bacterial pathogens have obligate or facultative intracellular life cycles enabling them to invade the epithelium, survive, and replicate in multiple environments. Bronchial and alveolar epithelial cells are not naturally phagocytic. Rather, some bacteria carry virulence factors that promote their entry into these cells. Once inside the epithelial cells, pathogens encode mechanisms that subvert normal trafficking pathways such that they may replicate in a membrane bound compartment or the cytoplasm. In order to replicate extracellularly, organisms must escape the cell which typically involves lysis caused by bacterial pore-forming toxins, induction of apoptosis, or may simply be the result of massive replication. 

## 2. Pathogen Detection

Detection of conserved structural motifs, termed pathogen associated molecular patterns (PAMPs), is achieved by the expression of surface receptors, both on the cell surface and in endosomes. Detection of an invading bacterium through a particular PRR results in the production of proinflammatory cytokines and chemokines that recruit and activate immune effector cells, such as granulocytes and T cells, to the site of infection. In addition to mediating a localized inflammatory response, soluble PRRs and antimicrobial peptides can also directly mediate killing of invading organisms through disruption of the cell membrane, resulting in osmotic lysis of bacteria [14]. 

Toll-like receptors (TLR) are transmembrane proteins that form a major family of PRRs and are ubiquitously expressed. TLRs form homo- and heterodimers with other TLRs or accessory proteins that are together responsible for the recognition of a variety of PAMPs including bacterial lipoprotein, lipopolysaccharide (LPS), peptidoglycan, flagellin, RNA, and nonmethylated CpG DNA. Different complexes are thought to mediate specific signal transduction pathways allowing for an increased repertoire of downstream responses. These proteins signal both innate, and adaptive immune responses and their collective action is essential for immune defense against bacterial pathogens. TLR2, 4, and 5 detect most species of bacteria and each of these signal through the common adaptor protein MyD88, located on the cytoplasmic face of the plasma membrane. Signal transduction via phosphorylation cascade activates the nuclear translocation of NF-*κ*B, which leads to the production of proinflammatory cytokines and chemokines. In addition, MyD88-independent signaling can also occur, leading to the expression of type I interferons (IFN-I) which also activate expression of proinflammatory cytokines and chemokines [15]. 

Bacterial LPS is composed of lipid A, core polysaccharide, and O-antigen. Lipid A composition varies between bacterial species and between cells of the same species and plays an important role in the pathogen's ability to colonize the host lung. Recognition of lipid A is achieved by the delivery of monomeric LPS micelles to CD14 by soluble LPS-binding protein (LBP) [16]. TLR4, in complex with the accessory protein MD2, then associates with CD14 forming the TLR4-MD2-CD14 LPS receptor complex. Upon complex formation, the cytoplasmic tail of TLR4 is able to associate with MyD88 leading to NF-*κ*B activation. Alternatively, TLR4 can also signal through the adaptor TRIF/TRAM leading to the activation of the transcription factor IRF-3 and the subsequent expression of type I interferon [17, 18]. Both the MyD88-dependent and independent pathways are important for host defense in the lungs, as mice deficient in these processes are more susceptible to pulmonary infection by many, but not all, bacterial species. Moreover, each pathway plays a distinct role against specific pathogens. TLR4 signaling can be initiated either on the cell surface or from the cytoplasm where the receptor remains associated with pathogen containing endosomes. In addition, TLR4 signaling induces crosstalk with other PRRs. For example, TLR4 activation upregulates surface expression of TLR2, enhancing the capacity of cells to be activated in response to PAMPs [19].

TLR2 primarily responds to Gram-positive bacteria through the detection of lipoteichoic acid and peptidoglycan from the apical surface of airway epithelial cells [20]. TLR2 has several coreceptors, including CD14 and CD36, which likely lead to specific activation patterns. In addition, gangliosides, some of which function as receptors for pathogen invasion, can also act as coreceptors for TLR2 and alter its ability to respond to PAMPs. Like TLR4, TLR2 signaling can be MyD88-dependent or independent, resulting in NF-*κ*B or IRF-3 activation and can be activated from the cell surface or from intracellular compartments [21]. In addition to the toll-like receptors, other PRRs such as the nucleotide oligomerization domain (NOD)-like receptors (NLRs), sense intracellular pathogens through the detection of peptidoglycan and bacterial DNA in the host cell cytoplasm. Retinoid-inducible gene I (RIG-I) and melanoma differentiation-associated gene 5 (MDA5) are RNA helicases that recognize microbial RNA and methylated DNA in the cytoplasm and activate IRF-3 [22]. In addition, DNA activator of interferon regulatory factors (DAI) is a major cytosolic DNA sensor that also leads to the activation of IRF-3 [23–25]. Thus, bacterial DNA is a potent inducer of inflammation through its effect on IFN-I gene expression from epithelial cells as well as alveolar macrophages.

TLR2, 4, and 5 are the primary sensors of bacteria, and crosstalk occurs between other toll-like receptors that respond to viral infection. For example, viral activation of TLR3 not only stimulates production of proinflammatory cytokines in type II cells but also leads to upregulation of TLR2 and some, but not all, of its coreceptors allowing for enhanced detection of bacteria [26]. In contrast, TLR5 is downregulated following TLR3 activation thereby decreasing the ability to respond to bacterial flagellin, leaving an opportunity for bacterial coinfection.

In addition to impacting PAMP recognition, viral infection may also assist in the deterioration of the airway epithelium or in modulating immune responses, leading to increased susceptibility to secondary bacterial pneumonia caused by opportunistic pathogens [27]. Many viruses destroy mucus producing or ciliated cells. Alternatively, viral infection may activate cell death pathways that then influence the ability of epithelial cells to respond to secondary infection. For example, following influenza virus infection, the host recruits monocytes to the airways which contribute proapoptotic signals to type I epithelial cells [28]. The resulting decay of the alveolar barrier can be exploited by opportunistic pathogens now able to invade and grow within the air spaces. Furthermore, influenza infection also depletes the host of effector cells such as monocytes, macrophages, and natural killer (NK) cells [28, 29]. 

## 3. Pathogen Clearance and Resolution of Inflammation

Cytokines and chemokines produced by airway epithelial cells rapidly stimulate recruitment and activation of neutrophils, eosinophils, monocytes, dendritic cells (DCs), and NK cells which are capable of destroying invading bacteria [30–33]. By 6 or 7 days postinfection, T cells are also recruited by RANTES or IP-10 production (for Th1 cells) and IL-1*β* (for Th2 cells). Subsequently, airway epithelial cells receive signals from recruited inflammatory cells to increase production of defense mechanisms. For example, elastase is a bactericidal serine protease stored in granuoles and is secreted by activated neutrophils in response to infection [34]. Following release of the granules, bronchial epithelial cells respond to elastase by upregulating the expression of *β*-defensins [35]. 

Because inflammatory cell activity in the airway is likely to cause tissue damage and congestion that interferes with lung function, epithelial cells also mediate downregulation of inflammatory responses following bacterial clearance in order to protect the lung from unnecessary tissue damage. Production of glucocorticoids and other lipids, as well as anti-inflammatory cytokines, such as IL-10 and TGF-*β*, by airway epithelial cells help to downregulate inflammation [36]. Lysophosphatidic acid (LPA) is a mediator produced by epithelial cells that regulates expression of pro- and anti-inflammatory cytokines and chemokines. This phospholipid acts by blocking the binding of interferon response factor-1 (IRF-1) to DNA, thereby reducing its ability to stimulate expression of downstream proinflammatory genes. LPA can also induce expression of lipid mediators of inflammation as well as repair enzymes. Costimulatory signals influence downstream responses, for example, IFN-*γ* and TNF-*α* co-stimulation of bronchial epithelial cells results in LPA-mediated downregulation of the neutrophil chemokine CCL5/RANTES. As IFN-*γ* and TNF-*α* are induced by pathogens and accumulate in the bronchus, LPA production by epithelial cells prevents long-term recruitment of neutrophils to prevent unnecessary damage to the lung.

## 4. Bacterial Pneumonia

When these defense mechanisms fail to prevent bacterial infection, pneumonia rapidly develops. Bacterial pneumonia can be subdivided into community acquired and hospital acquired. The most common community and hospital acquired pneumonia is caused by *Streptococcus pneumoniae*, an extracellular, opportunistic pathogen whose virulence is primarily derived from the production of a capsule which allows the organism to adhere to mucosal tissues and evade the innate immune responses of the lung, including surfactants, complement, and phagocytosis [37]. Others, such as *Klebsiella pneumoniae* and *Acinetobacter baumannii*, primarily cause hospital acquired infections using similar strategies [38, 39]. In contrast, community acquired pneumonia can be caused by other pathogens that target the lung as a primary replicative niche using multiple virulence strategies. For example, *Francisella tularensis*, *Staphylococcus aureus*, and *Yersinia pestis* cause lung injury as extracellular pathogens through the production of secreted toxins, but employ an intracellular life cycle that relies on entirely different virulence mechanisms to invade the epithelium, evade innate immune detection, and establish a successful infection. These pathogens have in common the capacity to produce numerous modulators of detection, as well as virulence factors that serve multiple roles during infection. However, the mechanisms whereby these pathogens evade the onslaught of detection and destruction initiated by airway epithelial cells are very different. Because of their abilities to disable multiple levels of innate immunity in the lungs, we will discuss these three pathogens in greater detail.

### 4.1. *Francisella tularensis*

*Francisella tularensis* infection through tick transmission, wound infection, or inhalation results in a number of disease manifestations in humans such as oculoglandular, oropharyngeal, gastrointestinal, typhoidal, and pneumonic tularemia [40]. Several subspecies have been identified and have varying degrees of pathogenicity, with *F. tularensis* subsp. *tularensis* (type A strain) being the most lethal, and *F. tularensis* subsp. *holarctica* (type B strain) being less pathogenic. *F. tularensis* subsp. *holarctica* is the parent strain of the attenuated Live Vaccine Strain (LVS) which was established by serial passage in the laboratory and was used as a vaccine for many years. A third subspecies, *F. tularensis *subsp. *novicida*, rarely causes disease in humans but is highly virulent in mice and is also routinely used as a model system for *F. tularensis *research [41]. Important mechanisms of virulence and immunity have been elucidated in the attenuated *Francisella *strains, yet significant differences exist in the ability of the human pathogens to control inflammatory responses and cause disease.

Pneumonic disease develops as a sequela of systemic infection, or as a result of inhaling bacteria and is characterized as a lobar pneumonia [40]. Symptoms are typically nonspecific and include fever, headache, and muscle aches, but patients can also present more severe indicators such as chest pain, bloody sputum, and dyspnea [42]. If left untreated, mortality rates reach 30–60%. *Francisella *is a facultative intracellular pathogen that targets macrophages and epithelial cells, and this interaction is essential for virulence. Bacteria interact with a multitude of receptors and are taken up by alveolar macrophages following opsonization in a process termed “looping phagocytosis” [43–45]. Nascent *Francisella *containing phagosomes do not mature into phagolysosomes but instead are lysed, allowing the bacteria to escape to the cytoplasm and replicate [46–48]. Escape from the phagosome and intracellular replication is dependent on the *Francisella *pathogenicity island (FPI) which encodes a Type VI secretion system [49, 50]. Expression of FPI genes is dependent on the global virulence regulator MglA [51]. Organisms eventually reside in autophagous vacuoles termed *Francisella *containing vacuoles (FCV) [48]. High level intracellular replication is thought to ultimately lead to macrophage lysis and spread of extracellular bacteria [52].

The presence of IFN-*γ* significantly increases the resistance of mice to *Francisella *infection [53, 54]. IFN-*γ* signaling through the IFN-*γ* receptor stimulates macrophages to upregulate bactericidal effector function against all three *Francisella *strains [55]. In murine and human macrophages, IFN-*γ* inhibits the intracellular growth of *F. novicida *and LVS in an iNOS-independent (inducible nitric oxide synthase) manner, likely by preventing bacteria from escaping the phagosome thereby promoting lysosomal fusion [56–58]. In contrast, *F. tularensis* phagosomal escape is not inhibited by IFN-*γ*, and growth is instead restricted in the cytoplasm [59]. The outer membrane protein OmpC of *F. novicida* inhibits IFN-*γ* induced STAT1 phosphorylation in macrophages [60]. Although OmpC is conserved in the other *Francisella spp.*, further studies are needed to determine if OmpC also blunts IFN-*γ* signaling for the human pathogens. 

Similar to other Gram-negative bacteria, *Francisella* has an LPS structure that contains lipid A, core, and O-antigen domains that are important to the pathogenesis of the organism [16, 61]. *Francisella *lipid A does not elicit an inflammatory response through TLR4 due to a lack of LBP binding [62–65]. Structural comparisons of *Francisella* lipid A to those that are highly proinflammatory, such as found in *E. coli*, indicate that its dephosphorylated glucosamine backbone and tetra-acylation may not be recognized by LBP, thereby preventing TLR4 signaling [66–70]. Artificial stimulation of TLR4 pathways before and after *F. novicida* infection, or use of a strain altered in lipid A structure (*flmF2*, *flmK*, and *lpxF*) induces an inflammatory response that lowers bacterial burden in the lung and increases the survival of infected mice, illustrating the importance of avoiding LPS-dependent TLR4 activation for pathogenesis [70–72].

In addition, intact *Francisella *LPS core has been shown to be important in regulating cytotoxicity towards macrophages [73]. When core components are mutated, the bacteria are attenuated and hypercytotoxicity towards J774.A1 macrophages is observed and that results in attenuated virulence. Interestingly, core mutants are able to invade macrophages treated with cytochalasin-D, indicating that uptake can be independent of phagocytosis and suggesting the existence of a surface located receptor. Moreover, the enhanced cytotoxicity of core mutants is caused by intracellular bacteria and is independent of TLR4. 

LPS O-antigen polysaccharide also contributes to *Francisella* pathogenicity by modulation of complement C3 activity. C3 deposition occurs on the surface of all virulent *Francisella *strains, but surface located C3b is converted to C3bi [74]. While C3b leads to cell lysis and opsonization, C3bi is only opsonizing and greatly enhances phagocytic uptake [43, 45, 75]. C4b and factor H deposition occurs normally on the surface of bacteria, but the C5b-C9 membrane attack complex (MAC) does not form, resulting in resistance to complement-mediated lysis [76]. Altered O-polysaccharide structures (as occurs in the LVS strain), renders the bacteria sensitive to complement through the C3b pathway and results in attenuation of virulence [74].

O-antigen from *F. tularensis* and LVS forms a capsular polysaccharide around the organism [77]. Loss of this capsule results in increased serum killing, reduced intracellular replication and hyper cytotoxicity toward macrophages [77, 78]. Conversely, *F. novicida* does not produce an O-antigen polysaccharide capsule, yet it is still resistant to complement-mediated lysis suggesting that distinct mechanisms of complement resistance may exist in this related strain [74, 77]. Passive transfer of serum raised against bacteria lacking the O-antigen, or immunization of mice with such a strain, does not protect mice from challenge by wild type bacteria, indicating that the O-antigen is also important for the development of humoral immunity [79, 80]. 

In addition to resisting the downstream effects of complement deposition, *Francisella *has also acquired mechanisms to resist human *β*-defensins (hBD) [81]. hBD-1 and hBD-2 show minimal to moderate bactericidal activity, respectively, against LVS and *F. novicida*, but only at artificially high concentrations. Conversely, hBD-3, which has potent antimicrobial activity towards many microorganisms, kills *Francisella* effectively [82, 83]. hBD-1 is constitutively expressed by lung type II epithelial cells while hBD-2 and hBD-3 expression is inducible [84, 85]. Consequently, to evade hBD-3, *Francisella* suppresses its expression in type II cells [81]. The mechanism of this suppression and the bacterial virulence factor(s) involved remain unknown. 

Although *Francisella *has evolved a hypoinflammatory cell surface, mammalian hosts have coevolved methods to detect the organism through alternative mechanisms. To this end, it has been known for some time that TLR2 activation and MyD88-dependent and independent signaling as well as induction of the inflammasome can result in effective clearance of *Francisella *in mouse models, and crosstalk between these pathways is critical [62, 86–91]. TLR2 recognition of *Francisella* lipoproteins likely occurs in the phagosome and results in protection of mice from lethal infection [62, 87, 92, 93]. Cell based assays indicate that LVS decreases TLR2-induced inflammation by activating phosphatidylinositol 3-kinase (PI3K) and upregulating MAPK phosphatase-1 (MKP-1), resulting in suppressed proinflammatory cytokine production from infected macrophages [94]. Whether this also occurs in airway epithelial cells has not yet been determined.

Until recently, in fact, relatively little attention has been given to the role of alveolar epithelial cells in *Francisella *pathogenesis, but bacteria are able to invade these cells *in vitro *and *in vivo* [95]. To adhere to type II cells, LVS expresses FsaP (*F. tularensis *surface associated protein), which promotes tight association to epithelial cells *in vitro* and may also use type IV pili [96, 97]. Similar to macrophages, invasion into alveolar epithelial cells is dependent on a preformed bacterial surface structure, as live and dead bacteria are internalized in a manner indistinguishable from one another. Invasion requires cells to be competent in cytoskeleton rearrangement, as inhibiting microfilament and microtubule activity abrogates internalization suggesting that it may enter through endocytosis. Internalized bacteria initially colocalize with the early endosomal marker EEA1, then later with the lysosomal marker LAMP-1. Bacteria then escape this compartment and replicate in the cytoplasm [98]. Interestingly, type II pneumocytes are stimulated to produce proinflammatory cytokines *in vitro *by *F. tularensis*, and thus, it is unclear whether airway epithelial cells sense and appropriately respond to invading bacteria [99].

Upon pulmonary challenge with *Francisella*, proinflammatory chemokine production by type II pneumocytes, as well as induction of matrix metalloproteinase-9 (MMP-9) and subsequent breakdown of the extracellular matrix, recruits neutrophils to the sites of infection [99, 100]. However, recent evidence suggests that during transendothelial migration, neutrophils acquire a depressed inflammatory phenotype that prevents exogenous activation [101]. Artificial depletion or recruitment of neutrophils during infection has little impact on the outcome of disease, indicating that bacteria are either resistant to neutrophil effector function, other cell types are able to control the infection, or both [102]. Subsequent analysis has shown that LVS inhibits NADPH oxidase assembly, supporting the hypothesis that bacteria are resistant to neutrophil function [103, 104]. Multiple virulence genes are required for LVS to inhibit oxidative burst in human neutrophils, including a number of acid phosphatases as well as pyrimidine biosynthesis genes [105–107]. 

### 4.2. *Staphylococcus aureus*

*Staphylococcus aureus* is a Gram-positive, opportunistic human pathogen, a commensal of the skin that colonizes an estimated 30% of the population and is a leading cause of hospital acquired infection. More recently, in addition to antibiotic resistant strains, more invasive isolates have emerged due to numerous genes acquired by this constantly evolving pathogen [108, 109]. Staphylococcal endocarditis, pneumonia, and sepsis now pose significant threats to both healthy and immune compromised individuals. These strains, known as community associated methicillin resistant *S. aureus *(CA-MRSA), now predominate in the human population worldwide and are no longer limited to nosocomial infections. 

Innate immune recognition of *S. aureus* is largely achieved through TLR2-dependent recognition of lipoteichoic acids, lipoproteins, and peptidoglycan, both from the cell surface and on endosomes of antigen presenting cells and type II epithelial cells [110–112]. In addition, mice lacking MyD88 are more susceptible to *S. aureus *infection suggesting that MyD88-dependent TLR2 signaling results in productive induction of immune responses. However, deletion of TLR2 did not completely abrogate induction of cytokines in macrophages, while deletion of TLR4 had no effect. These results suggest that additional PRRs, other than TLR2 and TLR4, detect *S. aureus*. Recent evidence points to a role for NLRs in recognizing *S. aureus* in macrophages due to the action of pore-forming toxins [113]. Owing to this efficient recognition of bacterial PAMPs, virtually all humans carry antibodies that recognize and opsonize *S. aureus*, and normal human serum promotes uptake by neutrophils. However, this does not always lead to bacterial killing. Instead, neutrophils and other phagocytic cells can be destroyed by the invasive strains [114, 115]. TLR2 activation leads to a proinflammatory response that recruits neutrophils, monocytes, T cells, and B cells to the infection site. In many cases, this response can be enough to clear the infection. Additionally, *S. aureus* is susceptible to surfactant, and opsonization of bacteria by SP-A can promote bacterial clearance [116].

*S. aureus* encodes a number of adhesins that play important roles in the pathogenesis of pneumonia. Invasion of epithelial cells involves fibronectin-binding protein and adhesins that have been specifically linked with invasive strains of *Staphylococcus* [117]. Invasion of these cells may be at least in part responsible for the ability of the bacteria to persistently colonize, as intracellular bacteria often evade host immunity [118]. Following invasion of human type II epithelial cells, there is little cell death *in vitro* [119]. In the intracellular environment, *Staphylococci *upregulate genes involved in iron scavenging and virulence, including several exotoxins, while simultaneously downregulating surface expressed protein A and the virulence associated transcription factor AgrA.

Invasion of *S. aureus* into nonphagocytic cells was initially demonstrated in cultured mammary epithelial cells [120, 121]. These experiments established that intracellular bacteria could escape the endosome and induce apoptosis of epithelial cells. More recently, *S. aureus* was shown to survive inside activated neutrophils and induce pyroptosis following escape from the phagolysosome [115]. Invasion of epithelial cells can be accomplished through interactions between bacterial fibronectin binding protein and *β*1-integrins which promote Src protein-tyrosine kinase signaling to internalize the bacterium [122–124]. In addition, *Staphylococcus *adherence to epithelial cells is enhanced following viral infection which causes increased expression of host cell receptors such as ICAM-1 and downregulation of TLR2. This likely allows increased invasion and reduced NF-*κ*B activation by the airway epithelial cells. 

*S. aureus* invasion of type II cells activates IFN-I. This activation is caused by the recognition of the conserved, multifunctional, secreted virulence factor protein A through its repeated IgG-binding domains [125]. Activation of IFN-I leads to IL-6 and TNF-*α* production but instead of benefitting the host, IFN-I is detrimental during *S. aureus* infection. Mice lacking the IFN-I receptor, IFNAR, are more resistant to *Staphylococcal *pneumonia, a phenotype that correlates with a reduction in neutrophils and an increase in dendritic cells recruited to the lungs. This effect was found to be independent of the adaptor TRIF and IFN-*β*, while dependent on STAT-3. Thus, even though it is highly stimulatory through PRRs, *Staphylococcal* protein A leads to evasion of host immunity.

Protein A is a major virulence factor, secreted by all pathogenic strains and required for the development of pneumonia in murine models [126]. Protein A binds host immunoglobulin with high affinity, thereby preventing its activation of Fc-receptor signaling. Protein A has long been appreciated for its role in virulence due to this activity, as well as interfering with the opsonization of *Staphylococci*, blocking phagocytosis, and disabling complement fixation by the classical pathway. In addition, protein A binds with high affinity to TNF-R1, a receptor for the proinflammatory cytokine TNF-*α*, on bronchial epithelial cells which leads to the recruitment of neutrophils to the lungs [127]. All of these interactions are mediated through the repeated IgG binding domains [128]. Thus the bacteria use a single protein to both evade immunity and establish a replicative niche in the lungs from inside and outside of host cells.

Extracellular bacteria produce many pore-forming toxins that specifically target leukocytes, some of which are unique to more virulent strains of *S. aureus*, while others are common to all of them [129–131]. Hemolysin is a well-conserved toxin that plays important roles during respiratory *S. aureus* infection. Hemolysin insertion into the plasma membrane of target cells creates a pore that alters ion gradients and membrane integrity, triggering cell death. Upon forming a pore in the smooth muscle cells that drive peristalsis, calcium release reduces contraction, allowing *S. aureus* access to the epithelial layer. There, hemolysin induces release of calcium from type II cells, leading to upregulation of IL-6 and prostaglandin production and, subsequently, inflammation [132, 133]. High affinity binding of *α*-hemolysin to type II epithelial cells is achieved through protein-protein interactions with ADAM-10, which not only leads to cell lysis, but also initiates signaling events that result in disruption of focal adhesions in the epithelial layer [134]. Once the bacterium accesses the alveolar spaces, *S. aureus* establishes a replicative niche, rapidly forming bacterial colonies that appear resistant to host neutrophils and other inflammatory cells [126]. Thus the activity of hemolysin paradoxically triggers inflammation while promoting invasion.

Other exotoxins are epidemiologically linked to invasive CA-MRSA strains. For example, *β*-toxin of *S. aureus* binds syndecan-1, is internalized, then renders the host cell more vulnerable to other pathogen encoded toxins [135]. In addition, the toxin increases vascular permeability and edema in the lung which exacerbates lung injury and inflammation. Although not all pathogenic *S. aureus* strains carry *β*-toxin, there appears to be a correlation between enhanced capacity for respiratory infections and the presence of the *β*-toxin gene [108]. CA-MRSA strains express an additional virulence factor termed Panton-Valentine Leukocidin (PVL), a toxin that assembles into pore-forming octamers on the surface of host cells. Association between PVL expression and the pathogenesis of *Staphylococcal *pneumonia appears evident in the human population, but a definitive role in virulence in mouse and rabbit models of infection is controversial. When PVL is overexpressed, it appears to promote pneumonia in a mouse model [136]. However, when expressed at endogenous levels, PVL is not required for invasive *Staphylococcal* disease [137]. Nevertheless, PVL possesses potent membrane lysing activity on human neutrophils [138, 139]. In addition to this well-characterized activity, PVL also modulates signaling through TLR2 [140]. Purified PVL toxin is sufficient to cause TLR2- and CD14-dependent inflammatory responses in the lungs following intranasal inoculation, suggesting multiple roles for TLR2 in responding to *S. aureus* invasion of the lung [137].

### 4.3. *Yersinia pestis*

Similar to *F. tularensis, Yersinia pestis* is a Gram-negative coccobacillus and is naturally transmitted to mammalian hosts by an arthropod vector [141]. Transmission via fleabite results in bubonic plague that can spread from the lymph to the blood, where organisms can then reach the lungs and cause secondary pneumonic plague. Once in the lung, organisms can be spread from person to person via aerosol droplets resulting in primary pneumonic plague, an acute bronchopneumonia. Pneumonic plague presents as a biphasic disease in that during the first 24–36 hours of infection little inflammation is observed. The lung environment then abruptly turns proinflammatory, accompanied by rapid bacterial growth and tissue necrosis [142]. Pulmonary infection ultimately results in a patchy bronchopneumonia containing necrotic lesions composed of fibrin, neutrophils, and bacterial colonies [143]. In humans, symptoms include fever, headache, weakness, bloody sputum, and dyspnea. If left untreated, the infection is nearly always fatal. 

*Y. pestis *has acquired mechanisms to modify its LPS structure in response to temperature which prevents recognition of the bacterium by TLR4. When grown at lower temperatures (21–27°C), *Y. pestis *expresses a mixture of tri-acyl, tetra-acyl, penta-acyl, and hexa-acylated lipid A structures which may be beneficial for growth in this environment [144, 145]. However, when grown at the mammalian body temperature (37°C), tri-acyl and tetra-acyl lipid A structures predominate, with no detectable hexa-acylation. Consequently, LPS isolated from bacteria grown at 37°C does not stimulate TLR4, and NF-*κ*B is not activated in human inflammatory cells, thereby delaying production of TNF-*α* and IL-8 [146–148]. LPS isolated from bacteria grown at 37°C also inhibits TLR4 activation. Shown in mixing experiments, *Y. pestis* LPS from bacteria grown at 37°C can suppress TLR4 activation elicited from normally proinflammatory LPS [149]. Similar results are seen in dendritic cells, where it has also been shown that tetra-acylated LPS inhibits cell signaling through TLR2 and TLR9 and inhibits upregulation of the Costimulatory molecules MHC-II, CD40, and CD86 [150]. Together, the data demonstrate that LPS modulates TLR signaling through multiple mechanisms and is of central importance to *Y. pestis* virulence.

*Yersinia pestis* also uses a type III secretion system (T3SS) to control inflammatory responses during infection through the injection of *Yersinia *outer proteins (Yops) into the host cell cytosol. Injection of Yops blocks phagocytic uptake by neutrophils, macrophages, and dendritic cells and ultimately causes host cell death [151–153]. This effect has historically been attributed to interactions with macrophages and neutrophils, however, the T3SS is active against epithelial cells and lymphocytes *in vitro*, suggesting that it is positioned to play multiple roles *in vivo*. Mutants that lack the T3SS are avirulent in pneumonic plague models, where they fail to evade early innate immune responses in the lung [154, 155]. Depletion of TNF-*α* but not IL-1*β* causes an increase in sensitivity of mice to *Y. pestis* lacking YopH, but not wild type bacteria, suggesting that YopH may impact the ability of the host to induce NF-*κ*B responses. Another type III effector protein, YopJ, has long been known to affect NF-*κ*B responses in macrophages. Recently, YopJ was shown to have similar activity when injected into bronchial epithelial cells where it reduced NF-*κ*B regulated gene expression, suggesting that this virulence factor may help prevent unwanted inflammatory responses during the early stages of infection [156]. However, YopJ is relatively dispensable for virulence during pneumonic plague, suggesting that additional mechanisms for suppressing NF-*κ*B regulated genes in alveolar macrophages and epithelial cells dominate during infection [157].

Effective T3S into phagocytic and epithelial cells has been shown to be dependent on the adhesive properties conferred by membrane proteins. Three proteins have been identified in *Y. pestis* that contribute this activity: Ail, Pla, and Psa. Ail (attachment-invasion locus) mediates binding to fibronectin, a component of the extracellular matrix [158]. Pla (plasminogen activator) has proteolytic and adhesive properties that also mediate binding to the extracellular matrix and perhaps other receptors on alveolar macrophages and dendritic cells [159–161]. Psa (pH 6 antigen) fimbriae bind to phosphatidylcholine, a component of cell membranes and surfactant, and to *β*1-linked, galactosyl-linked residues in glycosphingolipids [162, 163]. Psa appears to be more important for binding alveolar epithelial cells than to macrophages indicating that it may play a central role in penetration of the airway epithelium [164]. However, Psa is relatively dispensable for virulence during pneumonic plague, whereas Pla and Ail are essential [164]. Loss of all of these factors markedly reduces Yop-induced cytotoxicity towards target cells and attenuates virulence [165–168]. 

*Y. pestis* has a rough LPS structure and does not synthesize an O-antigen domain. Instead, bacteria utilize the multifunctional proteins mentioned above to resist killing by host antimicrobial molecules. Ail is highly expressed on the bacterial membrane at 26°C and 37°C and confers resistance to complement-mediated killing by serum derived from humans, rats, rabbits, sheep, goats, and guinea pigs but is dispensable for resistance to mouse serum [169]. Accordingly, strains lacking *ail* are highly attenuated in a rat model of pneumonic plague while in mice, *ail *mutants result in an increase in mean time to death, perhaps indicating a role for adhesion and internalization *in vivo* [170]. In addition, antimicrobial peptides such as cathelicidin and *β*-defensin have antimicrobial activity against attenuated strains of *Y. pestis in vitro*, and expression of the surface located virulence factors Pla and CaF1 (Capsular protein F1) influence susceptibility to these peptides [171]. Pla is a serine protease with broad spectrum activity that plays an essential role in the development of pneumonic plague. Pla-catalyzed cleavage of cationic antimicrobial peptides provides a mechanism whereby *Y. pestis* can cleave and inactivate CAMPs [171]. Paradoxically, expression of CaF1, which forms antiphagocytic pili on the *Y. pestis* cell surface at 37°C, can reduce the protective effects elicited by Pla, likely through steric interference or alteration of substrate specificity. Pla may also directly mediate serum resistance due to its proteolytic activity on C3 [160]. 

*Yersinia* species are thought to be capable of invading epithelial cells through interactions between one or more adhesins and host cell *β*1 integrins [172]. At least three pathways of invasion have been suggested based on interactions between enteropathogenic *Yersiniae* and epithelial cells, the most efficient of which, mediated by the protein Invasin, is not likely not to occur in *Y. pestis* because this gene is not expressed [173]. Nevertheless, invasion of the bronchial or type II alveolar epithelial cells is an attractive model by which *Y. pestis* would successfully penetrate the epithelial barrier without causing inflammation at early stages of infection. Alternative mechanisms for how bacteria invade the epithelium involve the action of one or more toxins produced by extracellular bacteria. In support of this model, Pla is required for *Y. pestis* to invade the lung parenchyma, suggesting that it may have a role in enhancing penetration of the alveolar epithelium [168].

Even under conditions that support high level, simultaneous expression of virulence factors that suppress phagocytosis and contribute to the extracellular lifecycle of *Y. pestis*, a small percentage of bacteria are engulfed by phagocytes *in vitro* [174]. Intracellular bacteria are equipped to resist antimicrobial activity and proliferate even in IFN-*γ* activated macrophages [175, 176]. Once phagocytosed by macrophages, bacteria prevent the acidification of vacuoles and begin replicating independent of the T3SS [177, 178]. Replication in activated macrophages requires the protein RipA which directly reduces NO levels without modulating iNOS expression [175]. Intracellular survival is also dependent on *phoPQ* (a two-component signal transduction system that responds to low [Mg^+2^]), *ugd*, *pmrK *(predicted phagosomal antimicrobial peptide resistance genes), and *mgtC* (a low-Mg^2+^ induced gene) which are important for the early intracellular survival of *Y. pestis * [179–181]. In addition, antibody opsonization of *Y. pestis* promotes phagocytic uptake, but the bacteria are not killed by macrophages and bacterial clearance by opsonizing antibodies requires neutrophils [182–184]. Together, the data suggest that survival in alveolar macrophages and perhaps also epithelial cells lining the airway may be an important virulence mechanism for invasive strategies utilized during pneumonic plague [164]. 

In addition to inhibiting bacteria from being internalized, T3S by extracellular bacteria also inhibits ROS production in phagocytic cells which is required to eliminate intracellular bacteria [153]. Neutrophils are resistant to Yop-induced apoptosis and inhibition of ROS production appears to prevent cells from undergoing phagocytosis-induced cell death (PICD), a mechanism used by neutrophils to contain infection and resolve inflammation [185, 186]. Thus, the data support a model whereby T3S blocking antibodies prevent Yop injection into neutrophils, allowing ROS production and subsequent killing of intra- and extracellular bacteria.

## 5. Conclusions

**Figure 1:**
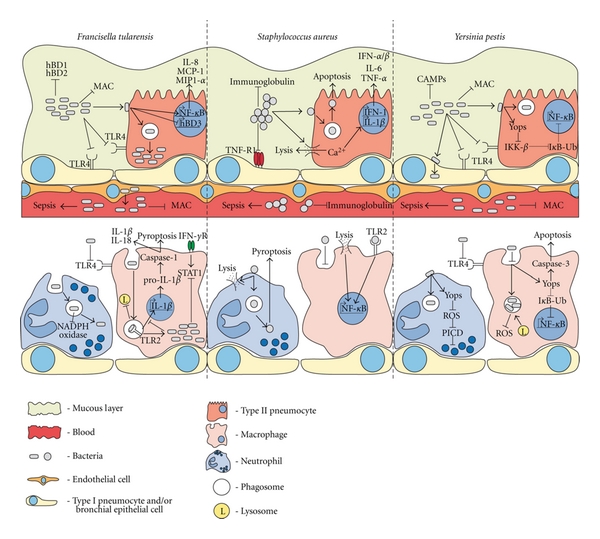
Disruption of airway defenses by *Francisella*, *Staphylococcus,* and *Yersinia*. Summary of host pathogen interactions used by these bacteria to modulate innate immune responses and invade the airway.

The mammalian lower respiratory tract is largely protected by the functions of airway epithelial cells. These cells are sentinels, orchestrating recruitment, activation and de-activation of inflammatory cells when microbes attempt to invade the lung. Through the continuous production of mucin and surfactant loaded with antimicrobial molecules, potentially harmful bacteria are trapped and cleared. If bacteria can avoid or resist these normally protective mechanisms, they need only to destroy or cross these cells to establish a replicative niche before an onslaught of inflammatory cells arrives. *Yersinia*, *Francisella,* and *Staphylococcus*, three bacterial pathogens with the capability to cause lower respiratory tract infection and acute pneumonia, possess multiple mechanisms for penetrating the epithelium and evading innate immunity, many of which exploit these defense mechanisms to promote virulence (Figure 1). In common between these and other bacterial pneumonias is the use of cell surface structures that evade recognition and resist the antimicrobial defenses of the airway epithelium. Bacterial pathogens have enormous capacity for continuous and rapid evolution allowing organisms to adapt in order to further tip the balance of host-pathogen interactions in favor of invasion across the epithelium, replication, and disease. 

## Figures and Tables

**Table 1:** Immunomodulatory roles of type II alveolar epithelial cells.

Role	Component	Function	Ref
*Antimicrobial*			
	Complement	Membrane disruption, opsonization, and inflammation	[6]
	Cathelicidin	Membrane disruption	[6]
	*β*-defensins	Membrane disruption	[6]
	Immunoglobulin	Complement-mediated lysis, agglutination, and opsonization	[6]
	Lipocalin 2	Iron sequestration	[6]
	Lysozyme	Membrane disruption	[6]
	Nitric oxide	Membrane disruption	[5]
	Surfactant	Agglutination (SpA, SpD), membrane disruption (SpB)	[6]

*Inflammation*			
	TLR2	Recognition of lipoproteins, lipoteichoic acid, and peptidoglycan	[6]
Receptors	TLR4	Recognition of lipopolysaccharide	[6]
	IL-2R	IL-2 receptor	[5]
	TNF-R1	TNF*α* receptor	[5]
	MHC-I	Antigen presentation to CD8 T cells	[6]
	MHC-II	Antigen presentation to CD4 T cells	[6]
Cytokines and chemokines	IL-1*α*	Prostaglandin production, induces TNF*α*	[6]
	IL-1*β*	Prostaglandin production, induces TNF*α*	[6]
	IL-4	Th2 Polarization, immunoglobulin production	[5]
	IL-6	T cell recruitment, B cell differentiation	[6]
	IL-8	Neutrophil chemotaxis	[6]
	GRO-*α*	Neutrophil chemotaxis	[6]
	ENA-78	Neutrophil chemotaxis	[6]
	MIP-2	Neutrophil chemotaxis	[6]
	TNF-*α*	Vasodilation, neutrophil activation	[6]
	GM-CSF	Granulocyte and monocyte differentiation	[5]
	RANTES	Monocyte and T cell recruitment	[6]
	MCP-1	Monocyte and T cell recruitment	[6]
	IFN-*α*/*β*	MHC-I Expression, NK cell activation	[6]
	IFN-*γ*	Th1 Polarization, macrophage activation	[5]
